# Dose and spatial resolution analysis of grating-based phase-contrast mammography using an inverse Compton x-ray source

**DOI:** 10.1117/1.JMI.7.2.023505

**Published:** 2020-04-22

**Authors:** Lisa Heck, Elena Eggl, Susanne Grandl, Martin Dierolf, Christoph Jud, Benedikt Günther, Klaus Achterhold, Doris Mayr, Bernhard Gleich, Karin Hellerhoff, Franz Pfeiffer, Julia Herzen

**Affiliations:** a Technical University of Munich, Chair of Biomedical Physics, Munich School of BioEngineering, Department of Physics, Garching, Germany; b Ludwig Maximilian University of Munich, Institute for Clinical Radiology, Munich, Germany; c Ludwig Maximilian University of Munich, Institute of Pathology, Munich, Germany; d Technical University of Munich, School of Medicine and Klinikum rechts der Isar, Department of Diagnostic and Interventional Radiology, Munich, Germany

**Keywords:** mammography, phase-contrast imaging, inverse Compton x-rays, radiation, spatial resolution

## Abstract

**Purpose:** Although the mortality rate of breast cancer was reduced with the introduction of screening mammography, many women undergo unnecessary subsequent examinations due to inconclusive diagnoses. Superposition of anatomical structures especially within dense breasts in conjunction with the inherently low soft tissue contrast of absorption images compromises image quality. This can be overcome by phase-contrast imaging.

**Approach:** We analyze the spatial resolution of grating-based multimodal mammography using a mammographic phantom and one freshly dissected mastectomy specimen at an inverse Compton x-ray source. Here, the focus was on estimating the spatial resolution with the sample in the beam path and discussing benefits and drawbacks of the method used and the estimation of the mean glandular dose. Finally, the possibility of improving the spatial resolution is investigated by comparing monochromatic grating-based mammography with the standard one.

**Results:** The spatial resolution is constant or also higher for the image acquired with monochromatic radiation and the contrast-to-noise ratio (CNR) is higher in our approach while the dose can be reduced by up to 20%.

**Conclusions:** In summary, phase-contrast imaging helps to improve tumor detection by advanced diagnostic image quality. We demonstrate a higher spatial resolution for one mastectomy specimen and increased CNR at an equal or lower dose for the monochromatic measurements.

## 1 Introduction

According to the World Health Organization, cancer is the second leading cause of death globally with 9.0 million deaths of all noncommunicable diseases deaths, where breast cancer is the most common type of cancer under the age of 60 worldwide.^1^^,^^2^ As a result of this, the reliable early detection of breast cancer is an important prerequisite for effective treatment. Several studies underline the successful introduction of the mammography screening program.^3^^–^^5^ But values such as the specificity (between 78% and 95%) and the sensitivity (between 69% and 94%) reveal the need of improvement of this imaging technique.^6^^,^^7^ One of the main issues that also leads to low values in the above-mentioned criteria is the superposition of anatomical structures within the breast especially for women with dense breast tissue. In those cases and due to the low soft tissue contrast, palpable masses or other suspicious findings are not always detected in the standard mammography screening.

Different approaches exist that are capable of improving standard mammography in terms of image quality and diagnostic image content. First, the brilliant monochromatic radiation provided by the synchrotron facilities can be exploited. This has the advantage that x-ray photons are eliminated that mainly contribute to the applied mean glandular dose (MGD) but not to image contrast. As a consequence, monochromatic radiation enables increasing the spatial resolution while the MGD is reduced. In breast imaging, the spatial resolution is an important factor especially for the detection of microcalcifications where a high resolution depending on several different aspects^8^ is desirable. Several published studies show the successful application of propagation-based phase-contrast mammography at synchrotrons with special focus on dose and improved diagnostic image content.^9^^–^^13^ However, this approach benefits from increasing spatial resolution and reducing dose but suffers from high costs, limited availability, and high infrastructure requirements. The second approach deals with the improvement of the soft tissue contrast to overcome the weak absorption contrast in conventional mammography, which can be improved by the above-mentioned image modalities or by grating-based phase-contrast imaging, which provides three signals simultaneously: The attenuation-based, the differential phase, and the dark-field signal.^14^^,^^15^ Several studies about grating-based phase-contrast mammography underline the benefit of this approach for the detection and classification of microcalcifications with the dark-field image^16^^–^^18^ and for improved soft tissue contrast with the phase-contrast image.^19^^–^^24^

Another way to overcome the current limitations of projection-based mammography is to expand to computed tomography. Several previous studies published the benefits and the technical feasibility of three-dimensional imaging of the breast.^25^^–^^28^ The latest work of Kalender et al.^29^^,^^30^ showed the clinical dose compatibility for this imaging method. They recently presented the first results of clinical *in vivo* imaging.^31^ As already mentioned, the attenuation-based signal suffers from low soft tissue contrast that should be improved by phase-contrast imaging. But for this application, the applied MGD remains a challenging topic that needs to be investigated and reduced for tomography applications of the breast as–for grating-based phase-contrast imaging–it is above the maximum allowable clinical MGD and only investigated with small but representative tissue sections of a mastectomy specimen.^32^^–^^34^ In contrast to that, other studies using the propagation-based phase-contrast approach have already successfully shown the feasibility of low-dose breast-computed tomography with synchrotron radiation.^35^^–^^38^ Therefore, the analysis of the applied MGD within this work and its possible dose reduction is of fundamental importance and represents some kind of preliminary work for grating-based phase-contrast-computed tomography of the female breast.

In order to further enhance contrast in x-ray breast imaging at lower MGD and higher spatial resolution compared to conventional mammographic imaging, we combined dose-compatible grating-based phase-contrast mammography with a brilliant inverse Compton x-ray source (ICS). The evaluation of several mastectomy specimens from a clinical point of view has already been published by Eggl et al.^39^ In contrast to that, this work now describes in detail the dose and resolution determination for the x-ray source and discusses its benefits and drawbacks by analyzing the data. In this study, we wanted to directly compare the spatial resolution of different image modalities (grating-based, clinical and monochromatic absorption-contrast images). Here, we followed the approach proposed by Modregger et al.,^40^ where a power spectrum analysis is performed on the acquired images in order to retrieve the spatial resolution in linepairs per millimeter (lp/mm). Increased spatial resolution is clinically relevant only as long as the applied dose remains at clinically applied levels. For assessing the MGD, it is important to take the different measurement parameters into account that affect the flux at the sample position. Correspondingly, this work presents an analysis of the MGD together with the estimation of the spatial resolution including a discussion of the advantages and disadvantages of the two chosen analysis approaches.

## 2 Materials and Methods

### 2.1 Study Protocol and Clinical Imaging

The mammography study was conducted with freshly dissected mastectomy specimens according to the Declaration of Helsinki and after the approval of the local ethics committee. After a detailed explanation of the study protocol, the patients gave their written consent before participating in the study. Before the experimental measurements and for a better comparison of the results, clinical *in vivo* and *ex vivo* images were acquired. The clinical *ex vivo* measurements were performed in a cranio-caudal (CC) or anteroposterior (AP) position with the specimen fixed in a metal-framed specimen holder to simulate breast compression. The clinical *in vivo* and *ex vivo* mammography images were both taken with a device by Hologic (Marlborough, USA) called Selenia Dimensions whose pixel size is $70\times 70\;\mu m^{2}$.

### 2.2 Working Principle of the MuCLS

**Fig. 1:**
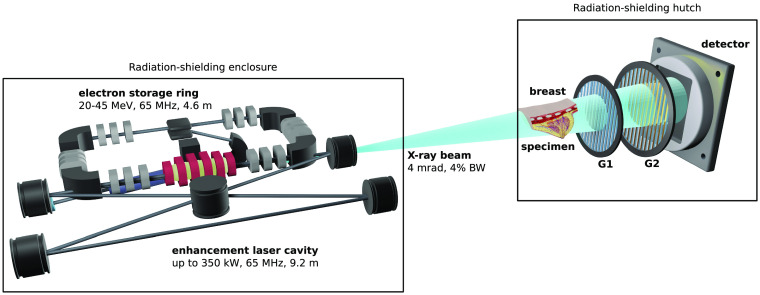
Schematic drawing of the experimental setup at the MuCLS (not to scale): the CLS is a storage ring-based ICS. Therefore, x-rays are generated by the collision of electrons accelerated to relativistic energies with a counterpropagating laser pulse stored in an enhancement cavity. The measurements were performed at an experimental station that is located about 15 m away from the interaction point.

The Munich Compact Light Source (MuCLS) is the first installation of an ICS focussing on x-ray applications. The compact light source (CLS, developed by Lyncean Technologies Inc., Fremont, USA) forms the MuCLS together with an imaging beamline developed in-house at the Technical University of Munich. A schematic drawing of the CLS and the experimental setup is shown in Fig. 1. Electrons are generated in a radiofrequency photocathode gun, accelerated to relativistic energies in the electron linear accelerator and injected into a small electron storage ring. A laser pulse is stored in an optical enhancement laser cavity.^41^^–^^43^ At the interaction point, where electrons collide head-on with the counterpropagating infrared laser pulse at a repetition rate of about 65 MHz, quasimonochromatic x-ray photons are produced with the x-ray energy $E_{x}$:

$$E_{x}\approx 4\gamma^{2}E_{L},$$ (1)

where $E_{L}$ is the energy of the laser photons and $\gamma =E_{e}/E_{0}$ is the ratio of the electron energy $E_{e}$ to the electron rest energy $E_{0}$. The x-ray energy $E_{x}$ of the MuCLS is tunable from 15 to 35 keV. The opening angle of the x-ray beam is 4 mrad, the x-rays are partially coherent and the flux is $3\cdot 10^{10}\;\mathrm{ph}/s$ for an x-ray energy of 35 keV with a horizontal and vertical r.m.s. source size of $50\times 50\;\mu m^{2}$.^44^ The beamline includes two experimental end stations. The measurements of this study have been conducted in the far end station, which is located 15 m away from the source point where the elliptic shape of the x-ray beam is $62\times 74\;{\mathrm{mm}}^{2}$ in size.^43^

### 2.3 Image Acquisition at the MuCLS

At the MuCLS, mammography images were acquired once with and once without the grating interferometer for a better comparison to the clinical images. For grating-based phase-contrast imaging, a Talbot interferometer was set up 16 m away from the source point of the MuCLS. The intergrating distance of the interferometer was about 25 cm. The periods of the phase grating and the analyser grating are 4.9  μm and 5.0  μm, respectively. The visibility of the interferometer was between 45% and 50%. The measurements were performed at an x-ray energy of 25 keV and with a Dexela 1512 flatpanel detector (PerkinElmer Inc., USA) equipped with a ${\mathrm{Gd}}_{2}O_{2}S$ scintillator with an effective pixel size of $71\times 71\;\mu m^{2}$. The breast specimen is fixated in the sample holder in order to keep its relative position in the holder constant, thereby ensuring comparability of all measurements. Due to the size of the sample, scanning the sample and stitching the images were required in order to obtain a full image of the breast specimen. The acquisition parameters are listed in Table 1 where the number of steps refers to the phase stepping of the grating-interferometer.

**Table 1:** Acquisition parameters whereby the exposure times always refer to the MGD in the column to the left. The exposure times are given for the whole acquisition including all steps and all stitching scans.

Sample	Energy (keV)	MGD mAC-Mx (mGy)	Exposure time (s)	MGD mgb-Mx (mGy)	Total exposure time (s)	Number of steps	Stitching
I	25	0.3	75	0.9	275	11	5×5
Phantom	25	1.0 to 2.0	40 to 80	0.7 to 1.8	28 to 72	7 or 9	2×2

### 2.4 Dose Calculation

The MGD of the clinically acquired mammography images is automatically registered by the imaging device. They are listed in Table 2 together with the x-ray tube settings and the compressed breast thickness. For the *ex vivo* measurements, the measured thickness was corrected for the contribution of the shape of the sample holder.

**Table 2:** Acquisition parameters of the clinical *in vivo* and *ex vivo* measurements.

Sample	X-ray tube settings	MGD civAC-Mx (mGy)	MGD cevAC-Mx (mGy)	Compressed thickness (cm)
I	30 kVp (W/Rh), 100 mAs	2.9	1.4 (AP)	4.5
Phantom	28 kVp (W/Rh), 200 mAs	—	2.0	4.5

For the MGD calculation, one needs conversion factors between air kerma K(E) and the MGD. Several conversion factors are tabulated in the literature, which are mostly used either in Europe^45^^–^^47^ or in the United States.^48^^,^^49^ These conversion factors are only tabulated for certain x-ray spectra but not for the MuCLS spectrum. As an alternative, the monoenergetic normalized glandular dose coefficients DgN(E) proposed by Boone et al. can be used to convert air kerma to MGD for any arbitrary spectrum. In addition, these coefficients depend on the breast thickness and the glandularity, which has been assumed as a 50%/50% distribution of adipose and glandular tissue for all samples. First, the air kerma K(E) has to be determined. Knowing the photon flux Φ(E) at the sample position, the energy-dependent air kerma K(E) for the known MuCLS spectrum^43^ can be calculated as^50^

$$K(E)=E\cdot \Phi (E)\cdot {\left[\frac{\mu_{\mathrm{en}}}{\rho }(E)\right]}_{\text{air}},$$ (2)

and depends additionally on the mass energy attenuation coefficient of air $\left(\mu_{\mathrm{en}}/\rho {)}_{\text{air}}(E\right)$.^51^ Considering the efficiency of the silicon sensor of the single photon-counting Pilatus 200 K detector (Dectris AG, Baden, Switzerland),^52^ which was used for the flux reference measurements, the x-ray spectrum, and the distance between detector and sample position, the photon flux per energy bin Φ(E) was calculated. The flux is defined as

$$\Phi =\frac{\frac{\text{Pilatus counts}}{{\mathrm{mm}}^{2}\;\text{frame}}}{QE_{\text{Pilatus}}\cdot T_{\text{air}}}=\frac{\text{Photon fluence}}{{\mathrm{mm}}^{2}\;\text{frame}}.$$ (3)

The transmitted intensity T can be obtained with

$$T=\frac{\overset{E_{\max}}{\underset{E=E_{\min}}{\sum }}S(E)\times \exp(-\frac{\mu }{\rho }\cdot \rho \cdot d)}{\overset{E_{\max}}{\underset{E=E_{\min}}{\sum }}S(E)},$$ (4)

where S(E) is the normalized intensity of the spectrum, μ/ρ is the materials absorption coefficient, ρ is the density, and d is the traversed thickness. The quantum efficiency for the Pilatus detector ${\mathrm{QE}}_{\text{Pilatus}}=1-T_{\mathrm{Si}}$ follows from this equation. To allow accurate calculation of the air kerma for each scan, the incident photon flux was recorded during measurements with a scintillation counter that had previously been cross-calibrated with the Pilatus detector. By summing up all energy bins E, the MGD can be calculated according to the following equation adapted from Boone et al.:^25^

$$\mathrm{MGD}=\underset{E}{\sum }K(E)(\mathrm{mGy})\cdot \kappa (\frac{R}{\mathrm{mGy}})\cdot DgN(E)(\frac{\mathrm{mGy}}{R}),$$ (5)

but modified with the commonly used unit air kerma K(E) instead of the older unit exposure since the adjusted equation for exposure given in Ref. 25 is incorrect.^53^ The corresponding conversion factor between the units Röntgen and Gray for the quantities exposure and air kerma is κ=0.114  R/mGy.

### 2.5 Analysis of the Image Quality

**Fig. 2:**
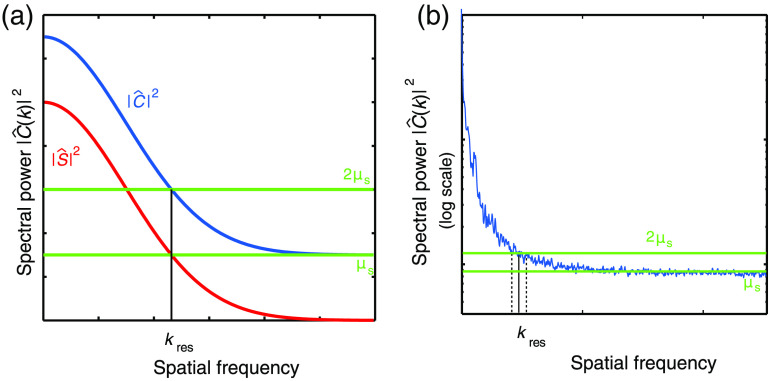
Spectral power analysis for the determination of spatial frequency: (a) Visualization of the resolution criterion. The maximal spatial frequency is defined as the frequency where the spectral power S of the signal s equals the spectral power of the noise $\mu_{s}$. In experimental data, only the total signal, including noise, is measured. The maximal spatial frequency is then given by the intersection of the spectral power C of the experimental signal c and the double of the spectral power of the noise baseline. (b) Example for the determination of the maximal spatial frequency on the experimental data. The smallest and highest spatial frequencies where the noisy spectra power curve intersects the $2\mu_{s}$ line is given by the black, vertical dotted lines. Then, the calculation of the mean value results in the actual spatial resolution of the image. Subfigure (a) adapted from Ref. 40.

This work focuses on the estimation of the spatial resolution with the sample in the beam path. This is an important point to consider in breast imaging, where microcalcifications have to be detected and distinguished. The spatial resolution of an image depends, among others, on the focal spot size, the sample movement, the scintillator thickness, and the pixel size.^8^ According to the Nyquist limit of 0.5 lp/px, the maximal achievable spatial resolution depends on the effective pixel size of the detector. In order to obtain the spatial resolution in linepairs per millimeter, an analysis of the power spectrum has been performed with the method proposed by Modregger et al.^40^ This approach is an objective criterion for the estimation of the resolution of an image with the sample in the beam path. For the estimation, the squared norm of the Fourier transformed images was calculated and afterward filtered with a Savitzky–Golay instead of a Gaussian filter as proposed by Modregger et al. to reduce large uncertainties. Then, the spatial resolution is determined by the maximal spatial frequency where the spectral power of the signal equals twice the spectral power of the noise baseline.^40^ In Fig. 2, a visualization of the spectral power analysis for the determination of the resolution is shown. In order to calculate the spatial resolution in linepairs per millimeter, the current unit of linepairs per pixel of the x axis has to be converted. Therefore, we take the conversion of the units from lp/px to lp/mm with a pixel size $x_{\text{detector}}$ from the detector, which is defined as

$$1\frac{\mathrm{lp}}{\mathrm{px}}=\frac{1\;\mathrm{lp}}{x_{\text{detector}}}.$$ (6)

In our case, with an effective pixel size of 71  μm for the Dexela detector and an effective pixel size of 68  μm for the clinical device, this results in the following unit conversion factors for the x axis of the plotted spectral power analysis:

$$\text{Dexela conversion}:\;1\frac{\mathrm{lp}}{\mathrm{px}}=\frac{1\mathrm{lp}}{71\;\;\mu m}=14.09\frac{\mathrm{lp}}{\mathrm{mm}}\;\text{Clinical conversion}:\;1\frac{\mathrm{lp}}{\mathrm{px}}=\frac{1\mathrm{lp}}{68\;\mu m}=14.71\frac{\mathrm{lp}}{\mathrm{mm}}.$$ (7)

In addition to the spatial resolution analysis, the contrast-to-noise ratio (CNR) is defined as the difference of two average signals $\overset{¯}{s_{1}}$ and $\overset{¯}{s_{2}}$ divided by the standard deviation $\sigma_{\mathrm{BG}}$ within a region of interest of a background region:

$$\mathrm{CNR}=\frac{\left(\overset{¯}{s_{1}}-\overset{¯}{s_{2}}\right)}{\sigma_{\mathrm{BG}}}.$$ (8)

## 3 Results

This study comprises the measurements of a mammographic phantom and one freshly dissected mastectomy specimen. The breast specimen investigated in this study has a multicentric lobular invasive carcinoma (G2) and a lobular carcinoma *in situ* with a maximal tumor diameter size of 51 mm.

### 3.1 Spatial Resolution Analysis with a Mammographic Accreditation Phantom

**Fig. 3:**
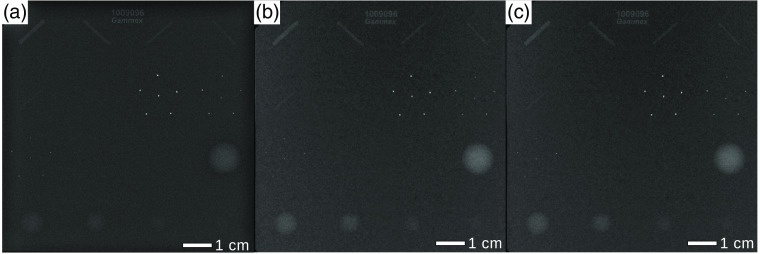
Absorption-only radiographs of the mammographic accreditation phantom: (a) clinical mammography (cevAC-Mx) with an MGD of 2.0 mGy, (b) mAC-Mx with an MGD of 2.0 mGy, and (c) with an MGD of 1.6 mGy. All images were scaled for maximum detail visibility.

**Fig. 4:**
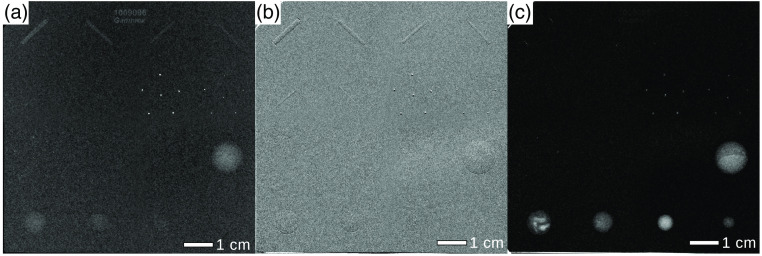
Monochromatic grating-based phase-contrast imaging showing (a) mgbAC-Mx, (b) mgbDPC-Mx, and (c) mgbDFC-Mx for an MGD of 1.8 mGy. All images were scaled for maximum detail visibility.

This section presents a quantitative analysis based on the calculation of the spatial resolution (as described in Sec. 2.5) and the CNR of a mammographic accreditation phantom (Mammo 156™ Phantom, Sun Nuclear Coorporation, Middleton, USA). The spatial resolution and the CNR of the monochromatic measurements at the MuCLS, both with and without grating interferometer, are compared to those of the measurements at the conventional mammography device. Images at the MuCLS were acquired with different exposure times whereas the clinical images are taken with the automated exposure control of the clinical device. The results for the classical absorption measurements are shown in Fig. 3 and the grating-based images in Fig. 4. The results of the analysis of the CNR for the different test objects (Nylon fibrils, simulated microcalcifications, and tumor-like masses) and of the spatial resolution are listed in Tables 3 and 4, respectively.

**Table 3:** CNR calculated for a quantitative analysis with the mammographic accreditation phantom. The numbers have been chosen so that the small numbers refer to the largest structures and the large numbers to the smaller structures.

Modality	MGD (mGy)	Fibers		Calcifications		Tumor masses	
		1	4	1	3	1	5
cevAC-Mx	2.0	2.51	1.99	36.84	13.89	5.92	0.53
mAC-Mx	1.0	3.16	0.11	30.86	11.94	8.64	1.50
mAC-Mx	1.6	3.51	1.61	38.47	15.60	10.70	2.73
mAC-Mx	2.0	4.71	1.39	44.11	16.85	12.19	2.67
mgbAC-Mx	1.8	4.46	0.15	25.32	13.25	7.14	0.89
mgbDFC-Mx	1.8	0.65	0.16	6.50	10.42	15.00	9.59

**Table 4:** Spatial resolution calculated with an analysis of the noise power spectrum.

Sample	cevAC-Mx (lp/mm)	mAC-Mx (lp/mm)	mgbAC-Mx (lp/mm)	mgbDFC-Mx (lp/mm)
I	2.06±0.34	2.90±0.29	3.26±0.05	4.13±0.08
Phantom	3.22±0.18	3.32±0.01	3.35±0.11	4.12±0.11

According to the American College of Radiology (ACR), a minimum of four fibrils, three groups of microcalcifications, and three tumor masses have to be resolved for the standard criteria of clinical image quality.^54^ This criterion is met by the following absorption-contrast images: clinical *ex vivo* absorption-contrast mammography (cevAC-Mx) [cf. Fig. 3(A)], monochromatic absorption-contrast mammography (mAC-Mx) [cf. Figs. 3(B) and 3(C)], and monochromatic grating-based absorption-contrast mammography (mgbAC-Mx) [Fig. 4(A)]. The comparison of the absorption-only radiographs without gratings in the beam in Fig. 3 reveals that on the one hand, the mAC-Mx at the same MGD slightly outperforms the cevAC-Mx image in terms of the CNR for almost all structures (except fiber structure 4). On the other hand, the MGD can be reduced by up to 20% while retaining the same CNR as in the cevAC-Mx image. Comparing the calculated spatial resolution of the mammographic accreditation phantom and considering their uncertainties, the spatial resolution of the monochromatically taken images for the absorption-contrast imaging does not exceed the cevAc-Mx image. It can also be noted that the spatial resolution of the dark-field image is higher than that of the absorption-based images. This makes sense since the dark-field image is sensitive to scattering at material interfaces, thus tending to emphasize edges more and thus sharpening the image.

In Fig. 4, the images of mgb-Mx are presented for an MGD of 1.8 mGy. At a slightly lower MGD for Figs. 4(a)–4(c) compared to cevAC-Mx in Fig. 3(a), the monochromatic grating-based results outperform the clinical device in terms of the CNR for tumor masses and big fiber structures but not for the microcalcifications (cf. Table 3). Furthermore, we can not calculate a CNR and perform a quantitative analysis of the monochromatic grating-based differential phase-contrast mammography (mgbDPC-Mx) images due to the differential nature of the signal. However, it can be seen that all six fiber structures are detectable in mgbDPC-Mx, which is not possible in any other image modality (absorption or dark-field contrast). Taking the calculated spatial resolution into account, one notices that the spatial resolution of the monochromatic grating-based dark-field-contrast mammography (mgbDFC-Mx) image is lower than in the clinical absorption-contrast image. All in all, grating-based imaging benefits from the simultaneous availability of absorption-, differential-phase and dark-field contrast, thereby exceeding the ACR criteria.

### 3.2 Spatial Resolution Analysis on a Mastectomy Specimen

**Fig. 5:**
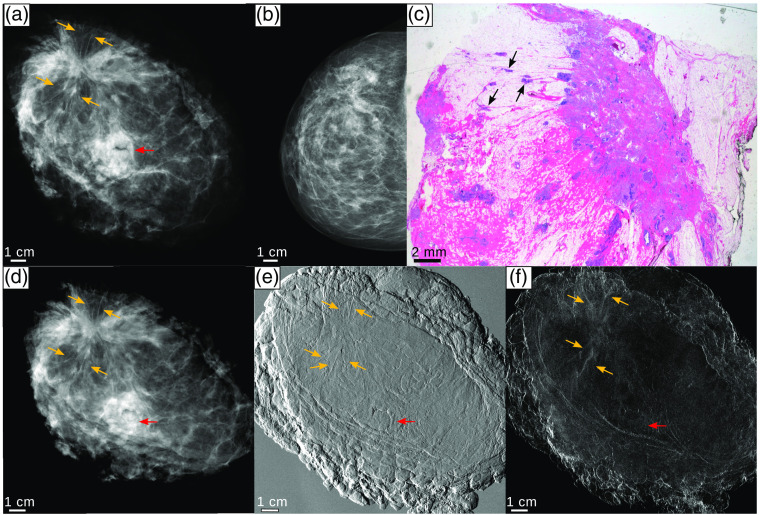
Advanced detection of tumor lesions. (a) The cevAC-Mx image in AP position, (b) civAC-Mx image in crandio-caudal position, and (c) the histopathological analysis of the sample are depicted in the first row. The second row presents the results acquired with grating interferometry at the MuCLS including (d) the mgbAC-Mx, (e) the mgbDPC-Mx, and (f) the mgbDFC-Mx images. All images in the second row are taken in AP position The red arrow depicts the mamilla, whereas the orange arrows in the radiographs and the black arrows in the histopathology image depict the cancerous lesions. All images were scaled for maximum detail visibility.

The specimen presented here was chosen because it incorporated tumorous lesions and thus allowed demonstrating their improved detection with the grating-based phase-contrast imaging. The results are shown in Fig. 5. The first row, Figs. 5(a)–5(c), displays the clinical and histology images and the second row, Figs. 5(d)–5(f), the monochromatic images. The clinical history has shown the following in the right breast: A palpable mass and also skin retraction that has been verified by clinical *in vivo* absorption-contrast mammography (civAC-Mx) where an asymmetry is visible in the respective region. In Figs. 5(d)–5(f), the mgbAC-Mx, the mgbDPC-Mx, and the mgbDFC-Mx images of the mastectomy specimen are presented. Those measurements have been performed in AP orientation, whereas the civAC-Mx image in Fig. 5(b) was measured in CC position. The red and orange arrows indicate the mamilla and the tumor lesions, respectively. Underlined by the calculated values presented in Table 4, the spatial resolution of the monochromatically acquired image modalities exceeds that of the cevAC-Mx. The resolution of the mgbDPC-Mx image was not calculated due to the differential nature of the signal. In addition to the increase of spatial resolution, an improved delineation of the tumor lesions is also possible with monochromatic grating-based phase-contrast imaging. The mgbAC-Mx image provides improved detection of cancerous lesions over the cevAC-Mx and civAC-Mx images. Fine tumor branches that originate from the carcinoma and perfusing into the surrounding tissue to both sides of the tumor are clearly visible in the mgbDPC-Mx image as indicated by the orange arrows. These tumor branches can also be identified in the mgbDFC-MX image but to a reduced extent. The histopathologic analysis after applying hematoxylin–eosin (H&E) staining proved the existence of the tumor spiculae originating from the main tumor [black arrows, Fig. 5(c)].

## 4 Discussion

This work presents grating-based phase-contrast and classical absorption mammography images of one breast specimen and a mammographic phantom measured at an ICS. The imaging performance of this approach is compared to clinical mammography. Thereby, this work focuses on the estimation of the spatial resolution with a sample in the beam path in linepairs per millimeter for mammographic application employing a power spectrum analysis (cf. Table 4). We successfully demonstrate a higher spatial resolution for the MuCLS measurements within one mastectomy sample for the absorption and dark-field contrast images at the same or reduced MGD compared to conventional mammography. A higher spatial resolution can be obtained with monochromatic radiation compared to conventional mammography for absorption and dark-field contrast images of one mastectomy specimen while keeping the MGD constant or even reducing it. However, for the mammographic accreditation phantom, the spatial resolution of all images agrees within their uncertainties. Thus, we achieved nearly the same spatial resolution for all the absorption-contrast images of the phantom but no significant improvement. In order to further decrease the uncertainties, we have applied a Savitzky–Golay instead of a Gaussian filter since the value of the spatial resolution would be more exact without large uncertainties. The spatial resolution is mainly influenced by two different factors: On the one hand, it depends on the source spot size of the imaging device. On the other hand, due to the limitation of the Nyquist-frequency of 0.5 lp/px, the spatial resolution depends on the effective pixel size of the setup, which was larger at the setup at the MuCLS (71  μm) than during the clinical measurements (68  μm). Consequently, the highest theoretically achievable spatial resolution for the clinical imaging system is 7.35 and 7.04  lp/mm for the experimental setup at the MuCLS. These two factors underline that the spatial resolution should mainly change by changing the imaging device or setup. Furthermore, cancerous lesions, which have been verified by histopathology, can be better detected in the differential phase-contrast image (cf. Fig. 5). The drawbacks and benefits of the dose and spatial resolution analysis will be discussed in the following. The calculation of the MGD depends on several different factors and is thus only an approximate estimation. Accuracy of this method is limited by the determination of the glandular dose coefficients DgN(E,t,g), which depend on the energy E but also on the thickness t and the glandularity g of the sample. Furthermore, the compressed breast thickness was measured when the breast was in the specimen holder whose contribution had to be subtracted. In addition, the glandularity was assumed to 50% adipose and 50% glandular tissue for both examined samples. Moreover, the quantum efficiency as well as the tabulated values of the absorption coefficient have uncertainties themselves that influence the accuracy of the calculation of the photon flux at the sample position. The uncertainty of the photon-counting Pilatus detector, which was used in this study, is 2%.^52^ All these factors have a significant influence on the MGD estimation and thus could lead to uncertainties in its calculation. In the framework of this study, the calculated air kerma is compared to air kerma values measured with a soft x-ray ionization chamber and the resulting uncertainties were ±10%. The calculated MGD calculation is therefore only an approximation. The dose analysis, which serves as a kind of preliminary work for breast-computed tomography, has shown that a dose reduction of about 20% is possible. Thus, grating-based phase-contrast breast-computed tomography has potential for preclinical studies in a dose-compatible range. The estimation of the spatial resolution is mainly based on the above-mentioned spatial resolution criterion.^40^ One drawback of this approach is the averaging of the high frequencies of the spectral power when the curve falls to a certain baseline. Thus, the estimation of the spatial resolution is mainly influenced by the setting of the flat profile of the noise baseline. Consequently, the estimation of the spatial resolution strongly depends on a previously defined noise criterion. In order to prevent this disadvantage in prospective spatial resolution analysis, another method has recently been proposed by Mizutani et al., which does not depend on defining a noise criterion.^55^ Thus, the spatial resolution strongly depends on the region that is chosen for averaging. However, this approach has several advantages: It can be applied to any type of image modality that is not of a differential nature. One is able to calculate a quantitative value for the spatial resolution with a sample in the beam path. This can be used to directly compare the spatial resolution of images acquired with different imaging devices. In conclusion, we demonstrate superior diagnostic image quality with a higher spatial resolution and an increased CNR at equal dose or equal diagnostic quality at lower dose for the monochromatic images compared to clinical ones.
